# “Transforming Pain”: Evaluation of a Multicomponent Workshop for the Treatment of Chronic Pain—A Quasi-Experimental Design with Control Group

**DOI:** 10.3390/healthcare14010108

**Published:** 2026-01-02

**Authors:** María Victoria Ruiz-Romero, María Begoña Gómez-Hernández, Ana Porrúa-Del Saz, María Blanca Martínez-Monrobé, Natalia Gutiérrez-Fernández, Almudena Arroyo-Rodríguez, Rosa Anastasia Garrido-Alfaro, Ángela C. López-Tarrida, Néstor Canal-Diez, María Dolores Guerra-Martín, Consuelo Pereira-Delgado

**Affiliations:** 1 San Juan de Dios del Aljarafe Hospital, Bormujos, 41930 Seville, Spain; mariavictoria.ruiz@sjd.es (M.V.R.-R.); mbghernandez@euef.comillas.edu (M.B.G.-H.); ana.porrua.delsaz@gmail.com (A.P.-D.S.); mbmmonrobe@euef.comillas.edu (M.B.M.-M.); nataliagutfer@gmail.com (N.G.-F.); rosagarridoalfaro@gmail.com (R.A.G.-A.); cmpereira@euef.comillas.edu (C.P.-D.); 2 San Juan de Dios Foundation, 28015 Madrid, Spain; aarroyor@comillas.edu; 3 Health Sciences Department, San Juan de Dios School, Comillas Pontifical University, Bormujos, 28036 Seville, Spain; 4 Student Research Hub José Bueno O.H., San Juan de Dios University Nursing Center, Bormujos, 41930 Seville, Spain; nesgercandie@gmail.com; 5 Department of Nursing, Faculty of Nursing, Physiotherapy and Podiatry, University of Seville, 41009 Seville, Spain; guema@us.es

**Keywords:** quality of life, self-care, resilience, anxiety, health outcomes

## Abstract

**Highlights:**

**What are the main findings?**

- Participants in the intervention group showed consistent and clinically relevant improvements across all outcomes compared with baseline: reductions in pain intensity and analgesic use, alongside increases in well-being, quality of life, perceived health, self-esteem, and resilience, as well as decreases in anxiety and depression.
- The core technique (mental analgesia) was associated with pain reduction in approximately three-quarters of participants, while around four-fifths reduced their medication intake, mainly by lowering the frequency of use or discontinuing certain drugs. Most participants also reported adopting healthier lifestyle habits.
- These benefits were sustained at medium-term follow-up, three months after completion of the workshop.

**What are the implications of the main findings?**

- Creating structured spaces where patients can share experiences with peers has therapeutic value, allowing individuals with different diagnoses but a common symptom, such as chronic pain, to connect, feel understood, and engage in mutual support.
- Assessing the combined use of the techniques, therapies, and tools applied in this program demonstrates their effectiveness in achieving the intended goal: transforming and alleviating pain by fostering active patient engagement in self-care, thereby improving quality of life and emotional well-being.

**Abstract:**

**Background/Objectives:** Between 20 and 30% of the global population experiences Chronic Pain (CP). A comprehensive, interdisciplinary approach incorporating non-pharmacological interventions and active patient participation is recommended. This study evaluated the short- and medium-term effectiveness of a multicomponent workshop compared with a control group. **Methods**: A detailed description of the workshop and a single-group before–after evaluation in 197 patients were recently published. The present study used a quasi-experimental before–after design with a three-month follow-up, comparing an intervention group (n = 64) with a contemporaneous control group that continued with usual care (n = 64). Validated scales were used to measure pain, well-being, quality of life (QoL), self-esteem, resilience, anxiety, and depression. Two ad hoc surveys assessed satisfaction and perceived impact on pain, medication use, habits, and mood. **Results**: A total of 128 patients participated (64 per group). The intervention group showed statistically significant improvements in all indicators at both short-term (end of workshop) and medium-term (three months) follow-up. Pain decreased by −1.3 (−3.0–0) [3 months: −1.0 (−3.0–−1.0)], anxiety by −3.0 (−5.0–−1.0) [3 months: −3.0 (−5.0–1.0)], and depression by −4.0 (−7.0–−2.0) [3 months: −3.0 (−6.0–0)]. Well-being increased by 3.0 (1.0–4.0) [3 months: 1.0 (0–4.0)]; QoL by 0.213 (0.072–0.388) [3 months: 0.185 (0.013–0.337)]; perceived health by 13.5 (0–30.0) [3 months: 10.0 (0–30.0)]; self-esteem by 4.5 (1.0–7.3) [3 months: 3.0 (−1.0–6.0)], and resilience by 1.0 (−1.0–5.0) [3 months: 1.0 (0.0–5.0)]. In the control group, resilience worsened (−1.0 [−5.0–1.0], *p* = 0.002) and depression increased (1.0 [−1.0–3.0], *p* = 0.037). Pain decreased in 47 participants (74.6%) at the end of the workshop [3 months: 34 (65.4%)]. Of 55 who used medication, 48 (81.4%) reduced their intake [3 months: 34; 68.0%]. Healthy habits improved in 58 (92.1%) [3 months: 40; 78.4%]. Mood improved: 26 (41.3%) described themselves as “cheerful” and 24 (38.1%) as “neutral” [3 months: 23; 44.2% and 14; 26.9%]. Overall satisfaction: 9.7 (scale 0–10). **Conclusions**: The workshop enabled patients to mitigate pain, actively participate in self-care, and improve quality of life, self-esteem, and emotional well-being. These effects persisted three months post-intervention.

## 1. Introduction

Chronic pain (CP) is defined as pain that persists or recurs for more than 3 months or that extends beyond the expected period of tissue healing. CP is currently conceptualized as a complex clinical condition involving interrelated sensory, emotional, cognitive, and social dimensions. It can be classified as chronic primary pain, when pain itself constitutes the principal clinical problem and is not better explained by another condition, or as chronic secondary pain, when it is attributable to a clearly identifiable underlying disorder [1,2]. Within the spectrum of musculoskeletal pain, low back pain continues to be the leading cause of disability worldwide. Recent global estimates converge in indicating that CP affects between 20% and 30% of the adult population worldwide [1,3,4]. In Europe, a 2024–2025 systematic review reported prevalence rates ranging from 20% to 33%, with a clear overrepresentation among women, older adults, and individuals with lower educational levels, as well as higher rates of somatic and mental health comorbidities [5]. The Global Burden of Disease study [6] estimated that 619 million people worldwide were living with low back pain in 2020 and projected an increase to 843 million by 2050, largely driven by population aging and growth. CP represents a major public health priority due to its high prevalence, long-term course, and its profound functional and psychosocial impact. Beyond the symptom of pain itself, the clinical profile of individuals suffering from CP is characterized by a high prevalence of anxiety and depression. A 2025 meta-analysis estimated that 40% of adults with CP experience both depression and anxiety, underscoring the need for systematic screening and integrated treatment approaches [7]. These figures imply that hundreds of millions of people live with CP that significantly impacts functionality, mental health, and productivity.

This scenario has consolidated a paradigm shift toward a biopsychosocial model that prioritizes non-pharmacological interventions as the first line of treatment, particularly for primary CP. The NICE Guideline NG193 (2021) [2], recommends structured exercise programs, cognitive-behavioral therapy, mind–body interventions such as mindfulness, and, in selected cases, acupuncture. Pharmacological treatments should be prescribed selectively, and the initiation of opioids for primary CP is discouraged due to their unfavorable risk–benefit profile.

A meta-analysis showed that programs combining exercise with psychological components can achieve greater improvements in pain-related outcomes than single-component strategies, although heterogeneity remains, and patient profiling and optimal therapeutic combinations require refinement [8]. Likewise, pain neuroscience education appears to yield more consistent benefits when integrated with exercise or other active components, particularly for psychosocial outcomes such as catastrophizing and kinesiophobia [9]. In parallel, recent meta-reviews suggest that e-health interventions and internet-based cognitive behavioral therapy can be effective options for musculoskeletal pain, especially when anxiety and depression are present, potentially improving access and scalability [10,11,12]. These findings align with results from multidisciplinary biopsychosocial rehabilitation and multicomponent workshops delivered in structured (often group-based) formats, which have demonstrated clinically meaningful benefits in conditions such as chronic low back pain and fibromyalgia [12,13,14,15,16].

Altogether, this growing body of evidence supports the need for non-pharmacological approaches delivered through multicomponent programs as a central element in CP management strategies. The San Juan de Dios Aljarafe Hospital (SJDAH), which provides healthcare to more than 300,000 residents across 28 municipalities, has been implementing multicomponent workshops for the management of non-oncological CP with non-pharmacological therapies since 2016. As of October 2025, a total of 25 workshop editions have been conducted, involving more than 400 patients. Recently, a detailed description of the workshop and an outcome evaluation were published, including patients who attended the workshop between November 2021 and May 2024; 197 patients completed the program and were assessed at 3 months (132; 67.0%) [17]. In the present study, patients who participated in the workshop between April 2023 and May 2025 and completed the 3-month follow-up (64 patients) are reported, and outcomes are compared with those of a contemporaneous control group that continued with usual care. Both within-group changes from baseline and between-group differences between the intervention and control groups are presented, and it is indicated whether the observed differences reached the MCID. The objective of the present study was to evaluate the short-term (at the end of the workshop) and medium-term (three months post-intervention) effectiveness of the program (improvements in pain, well-being, QoL, self-esteem, resilience, anxiety, depression, and medication use), compared with a control group that continued receiving standard treatment.

## 2. Materials and Methods

### 2.1. Study Design

This study followed a quasi-experimental, intra-group before–after design with a control group and follow-up assessments at one and three months.

This study evaluates the same multicomponent workshop previously described in Ruiz-Romero et al., Med. Sci. 2025, 13, 319 [17], but differs in design and aims: we introduce a contemporaneous control group and report comparative effectiveness at one and three months, whereas the prior study employed a single-group pretest–posttest design and focused additionally on healthcare utilization and medication counts.

### 2.2. Participants and Setting

Patients were referred via email or telephone by physicians from different specialties at SJDAH (mainly Rehabilitation, Traumatology, and Internal Medicine), by primary care physicians from the SJDAH reference area (Aljarafe Health District), and by CP patient associations. All referring professionals were previously informed in detail about the workshop’s characteristics, the type of patients for whom it was designed, its content, and its structure. Before being admitted to the workshop, patients were interviewed to ensure that they met the inclusion criteria.

Inclusion criteria: patients aged 18 years or older, diagnosed with non-oncological musculoskeletal CP for at least six months, whose pain persisted after at least six months of treatment with the analgesics prescribed by their physician, who voluntarily wished to participate in the workshops and the study, had signed informed consent, and completed the initial documentation. Exclusion criteria: patients in the diagnostic phase; those with pain exclusively related to oncological disease; individuals with severe cognitive or psychiatric disorders that prevented them from understanding the workshop content or the measurement instruments. Study dropouts: patients who missed two or more sessions and did not complete the evaluation documentation at the end of follow-up were considered losses.

All patients who participated in the study were on the waiting list for the workshop and met the inclusion criteria. Patients in the intervention group were those who successfully completed the workshop and additionally completed the follow-up questionnaires three months after its completion. Patients in the control group completed the questionnaires at the same time points as the workshop participants but did not receive the intervention; these patients continued with their usual pharmacological treatment.

The datasets from the previous study [17] and the present study are distinct. Patients included in this study were those who, in addition to completing the workshop, completed the 3-month follow-up after its completion and filled out all outcome measures currently included, as well as the patients in the control group, who also completed all outcome measures at the same time points as the intervention group.

### 2.3. Sample Size

Sample size was calculated based on the mean values of two main variables, pain and quality of life (QoL), selecting the larger value. The G*Power v3.1.9.6 software (Heinrich-Heine-Universität Düsseldorf, Düsseldorf, Germany) was used. An independent samples *t*-test was applied to compare the means of the two groups (control and experimental), with a significance level of 0.05 and statistical power of 0.80, yielding a sample size of 128 patients (64 per group). For intragroup before–after comparisons, a dependent samples *t*-test was used, with the same significance level (0.05) and power (0.80), yielding a sample size of 34 patients per group. Therefore, the minimum sample size required to meet the objectives of the quantitative study was 128 patients (64 in each group), allowing both intra- and intergroup comparisons.

Each workshop enrolled 20 patients, accounting for a 20% anticipated loss (patients missing two or more sessions), so approximately 16 participants were expected to complete each program.

### 2.4. Outcomes

The primary dependent variable was QoL; secondary dependent variables included: pain, well-being, anxiety, depression, self-esteem, resilience, mood, improvement of habits, and medication reduction and explanatory variables were: patient demographics, pain characteristics, prior pharmacological treatment, mood, and satisfaction with the workshop.

All questionnaires were self-administered by the participants and included: (A) Patient data form completed at the beginning of the workshop. (B) Patient-reported outcome measures (PROMs): self-assessment of the impact of the workshop on pain management through an ad hoc survey and validated scales. (C) Patient-reported experience measures (PREM): final evaluation of the workshop, overall satisfaction, and suggestions for improvement.

The validated scales used were:

- Pain intensity was measured using the Numeric Pain Rating Scale (0 = no pain, 10 = worst imaginable pain), a measure widely used in CP research and clinical practice. The minimally clinically important difference (MCID) is ≥2 points [18].
- Subjective well-being was assessed with the Numeric Well-being Scale (0 = worst possible well-being, 10 = best possible well-being), a single-item global indicator developed for clinical use in our program that mirrors the structure of the Numeric Pain Rating Scale to facilitate patient comprehension and minimize response burden. The most reasonable and defensible criterion is an improvement of ≥1.5–2 points [19].
- QoL was evaluated with the EuroQol-5D [20], which includes five dimensions (mobility, self-care, usual activities, pain or discomfort, anxiety or depression) and provides both an index value (0–1, with higher scores indicating better health) and a visual analogue scale for self-perceived health status (0–100). In CP populations, improvements of ≥0.05 of the index value are considered clinically relevant, and for the self-perceived health (“My Health”) (0–100), the accepted MCID is ≥7–10 points.
- Global self-esteem was assessed using the Rosenberg Self-Esteem Scale [21], scored on a 4-point Likert scale and yielding total scores from 9 to 36 in this study. There is no universal MCID for self-esteem, but longitudinal studies typically consider a difference of ≥2–3 points in the total score as a meaningful change.
- Resilience was measured with the Brief Resilience Scale [22], which comprises six items rated on a 5-point Likert scale (total score range: 6–30), with higher scores indicating greater perceived ability to “bounce back” from stress. In clinical reporting, a threshold of ≥1.5 points is commonly used to indicate a significant change.
- Anxiety and depressive symptoms were assessed with the Hospital Anxiety and Depression Scale [23], a 14-item instrument with two 7-item subscales (anxiety and depression), each scored from 0 to 21. The literature is quite consistent, placing the MCID between 1.5 and 1.7 points per subscale, and many trials use ≥2 points as the threshold for clinically relevant improvement.

### 2.5. Intervention

The intervention consisted of a group-based multicomponent workshop with a psychoeducational and self-care training focus, centered on pain control and emotional regulation. The approach encouraged active patient participation in the management of their condition, aiming to enhance health, well-being, and QoL.

The workshop was conducted by a multidisciplinary team comprising three physicians (specialists in Physical Medicine and Rehabilitation, Internal Medicine, and Preventive Medicine), a psychologist, a physiotherapist, and a nurse, all of whom were staff members at SJDAH (Bormujos, Seville, Spain). All professionals had specific training in the non-pharmacological management of CP, which qualified them to deliver psychoeducation and implement evidence-based, low-risk therapeutic strategies. Furthermore, patients who had previously completed the workshop in earlier editions participated as peer collaborators, sharing their experiences with newly enrolled participants. Between 15 and 20 patients participated in each group.

The multicomponent workshop incorporates a set of evidence-based, non-pharmacological strategies that are widely used in the management of CP. Relaxation techniques and guided self-healing meditations (such as mental analgesia and self-healing) fall within mindfulness-based and mind–body interventions, which have been shown to be effective in reducing pain intensity and alleviating emotional distress.

The workshop comprised five weekly sessions integrating relaxation, guided meditations, cognitive restructuring, acceptance-based strategies, and lifestyle optimization. Full details are available in Ruiz-Romero et al., Med. Sci. 2025, 13, 319 [17].

Patients were instructed to record daily the performance of three key activities (mental analgesia, self-healing meditation, and mirror affirmations), along with pain intensity and analgesic use. Each week, one or two additional home tasks were assigned and reviewed at the beginning of the following session. Patients from previous workshops were occasionally invited to share their experiences.

At the end of the workshop, participants received a toolkit guide summarizing the techniques learned to continue practicing them at home. A follow-up session one month after completion was conducted to reinforce learning and encourage ongoing application of techniques. During this meeting, both group-level and individual feedback reports were presented to each participant. At three months, follow-up assessments were repeated, and two additional sessions were offered, focused on self-esteem and motivation for sustained change.

### 2.6. Data Analysis

Data were analyzed using SPSS v27.0 (Statistical Package for the Social Sciences) (IBM Corp., Armonk, NY, USA). Qualitative variables (sex, education, employment status, family structure, etc.) were summarized as absolute (n) and relative (percentage) frequencies, and quantitative variables as medians and interquartile ranges (IQR), given their non-normal distribution. Comparisons between independent groups (intervention vs. control) were performed using the Chi-square test or Fisher’s exact test for categorical variables and the Mann–Whitney U test for quantitative variables. Within-group (before–after) comparisons in both the intervention and control groups were conducted using the Wilcoxon signed-rank test. Normality was assessed using the Kolmogorov–Smirnov test. Statistical significance was set at *p* < 0.05. The analyses were conducted using a per-protocol approach.

### 2.7. Ethical Aspects

The project was approved by the Research Ethics Committee (Code: 0213-N-22; 17 February 2022). All procedures complied with the ethical principles of the Declaration of Helsinki and Spanish Organic Law 3/2018 on the Protection of Personal Data. Personal data were not disclosed to third parties. The workshop is registered as a scientific work in the Territorial Register of Intellectual Property (No. 04/2024/3397, 1 March 2024).

## 3. Results

Patients included in the study were those who participated in the workshops conducted between April 2023 and May 2025, having completed the three-month follow-up questionnaires until reaching the minimum required sample size (64). Control groups were initiated in September 2024 and followed for the same period as the intervention group participants, concluding in August 2025, once the target sample (64) was achieved.

### 3.1. Baseline Characteristics of Participants

Table 1 presents the baseline characteristics of participants in each group and of the total sample. No statistically significant differences were found between the groups for any of the baseline study variables.

The median age of participants was 52.5 years (interquartile range: 46.0–57.0). 

A total of 115 (89.8%) were women. Regarding educational level, 29 (23.8%) had completed primary education, 42 (34.4%) had secondary or vocational training, and 32 (26.2%) held university degrees. Not require a caregiver 93 participants (73.8%); 86 (67.2%) had been absent from work. A total of 111 participants (86.7%) reported generalized pain (Table 1).

At baseline, 44 (34.6%) participants reported feeling discouraged, and 56 (44.1%) described themselves as depressed; Maximum pain intensity during the past month and the preceding six months was 9.0 (8.0–10.0); 110 (85.9%) used analgesics, and 87 (68.0%) took antidepressants, anxiolytics, or muscle relaxants; 68 (53.1%) had used morphine at some point (Table 1).

Baseline scores for measured scales were as follows: Pain 8.0 (6.0–8.0); Well-being 4.0 (2.0–5.0); QoL (EuroQol-5D) 0.354 (0.173–0.569); Self-perceived health 40.0 (25.0–50.0); Self-esteem 21.0 (19.0–25.0); Resilience 17.0 (12.0–19.0); Anxiety 13.0 (11.0–16.0); and Depression 11.0 (8.0–14.0) (Table 1).

The most frequent conditions were fibromyalgia (77; 31.7%), osteoarthritis (22; 9.1%), and low back pain (19; 7.8%); the most common pain locations were feet (65; 9.1%), hips (64; 9.0%), shoulders (55; 7.7%), pelvis (55; 7.7%), and hands (51; 7.2%) (Table 2).

### 3.2. Short-Term Results

At one month—corresponding to the end of the workshop for the intervention group—a statistically significant improvement was observed in all measured indicators and such differences reached or exceeded the MCID across all outcome except in two, pain and resilience. Pain decreased by −1.3 (−3.0–0) (MCID ≥ 2); anxiety by −3.0 (−5.0–−1.0) (MCID ≥ 2); and depression by −4.0 (−7.0–−2.0) (MCID ≥ 2); well-being increased by 3.0 (1.0–4.0) (MCID ≥ 2); QoL (EuroQol-5D index) by 0.213 (0.072–0.388) (MCID ≥ 0.05); self-perceived health (“My Health”) by 13.5 (0–30.0) (MCID ≥ 10); self-esteem by 4.5 (1.0–7.3) (MCID ≥ 3); and resilience by 1.0 (−1.0–5.0) (MCID ≥ 1.5). Conversely, the control group showed no statistically significant changes in any indicator, except for two that worsened: resilience –1.0 (−5.0–1.0) (*p* = 0.002) and depression +1.0 (−1.0–3.0) (*p* = 0.037) (Table 3).

### 3.3. Medium-Term Results

At three months after workshop completion (equivalent to four months from baseline in the control group), statistically significant improvement persisted across all measured indicators in the intervention group and these differences reached or exceeded the MCID for anxiety, depression, QoL, self-perceived health, and self-esteem, but not for pain, well-being, and resilience. Pain decreased by −1.0 (−3.0–−1.0) (MCID ≥ 2); anxiety by −3.0 (−5.0–1.0) (MCID ≥ 2); and depression by −3.0 (−6.0–0) (MCID ≥ 2); well-being increased by 1.0 (0–4.0) (MCID ≥ 2); QoL index by 0.185 (0.013–0.337) (MCID ≥ 0.05); self-perceived health (“My Health”) by 10.0 (0–30.0) (MCID ≥ 10); self-esteem by 3.0 (−1.0–6.0) (MCID ≥ 3); and resilience by 1.0 (0.0–5.0) (MCID ≥ 1.5). In contrast, no statistically significant differences were observed in the control group for any of the indicators (Table 4).

### 3.4. Comparison of Improvements Between the Intervention and Control Groups

The median changes in the different outcome measures relative to baseline in the intervention group were significantly different from the changes observed in the control group, both one month after initiating the workshop and at 4 months (Table 5).

### 3.5. Self-Assessment of the Workshop’s Impact

Of the 64 participants in the intervention group, 63 completed the PROMs self-assessment at the end of the workshop, and 52 repeated them at three months. A group of 47 (74.6%) participants reported a decrease in pain immediately after the workshop, and 34 (65.4%) maintained it at three months. Of the 55 who initially used analgesics, 48 (81.4%) reduced consumption (34; 68.0% at three months). A total amount of 58 (92.1%) patients reported improvement in their habits after the workshop (40; 78.4% at three months); while 26 (41.3%) described their mood as “cheerful” and 24 (38.1%) as “neutral” after the workshop; at three months, 23 (44.2%) and 14 (26.9%), respectively (Table 6).

Overall satisfaction with the workshop was 9.7/10, with the following sub-scores: recommendation level—9.8; clarity of content—9.6; relevance of activities—9.7; usefulness for pain management—8.9; and usefulness for managing the condition—8.9.

## 4. Discussion

The intervention group was associated with an improvement at the end of the workshop in all measured outcomes compared with baseline values. These improvements were statistically significant and consistent with other studies that have implemented non-pharmacological therapies. Pain decreased [24,25,26,27], as did the consumption of analgesics [25,27,28], while well-being [29,30], QoL [26,27,31], self-perceived health status, self-esteem, resilience, anxiety, and depression [24,27] all improved, and all outcome measures reached or exceeded the MCID, with the exception of pain and resilience.

The workshop’s core mental analgesia technique was associated with pain relief in roughly three out of four participants, and about four out of five reported reducing medication use, most commonly by taking doses less frequently or stopping specific drugs altogether. In the vast majority, it was also associated with improved lifestyle habits, as reported in other studies [32].

In contrast, no statistically significant differences were found in the control group for any of the indicators, except for resilience and depression, which showed a slight but significant deterioration.

In the medium term (three months after completion of the workshop), the intervention group was associated with improvements that were also observed in all indicators compared with initial values, and all outcome measures reached or exceeded the MCID, with the exception of pain, resilience and well-being. However, in the control group, no statistically significant differences were found in any of the indicators. This pattern suggests that the intervention was related to clinically meaningful benefits across most domains, while residual limitations in pain intensity, resilience and well-being may require longer follow-up or additional targeted strategies.

Furthermore, the intervention group reported continuing to manage pain using the workshop techniques in two-thirds of cases, and more than two-thirds maintained the reduction in medication use, with an increased number of participants who discontinued some drugs altogether, findings consistent with previous evaluations conducted by the research team [33]. Björnsdóttir et al. [26] also reported positive results up to six months, with reductions in pain and improvements in QoL; in other studies, however, these benefits were not sustained [34,35].

One distinctive feature of this workshop is the inclusion of patients with diverse conditions, mainly fibromyalgia, osteoarthritis, and low back pain. This diversity is considered enriching for participants, as it allows them to share experiences arising from different diseases but with a common symptom: CP, often accompanied by emotional suffering. In contrast, many multicomponent programs for CP target patients with a single diagnosis [12,27,31,36].

A major strength of the workshop lies in the combination of techniques, therapies, and tools applied. A similar experience has been developed in Catalonia, at the Parc Sanitari Sant Joan de Déu (Barcelona, Spain), led primarily by psychologists integrated within multidisciplinary teams, where psychological techniques constitute the core of the therapy. Researchers have evaluated several multicomponent interventions for people with fibromyalgia, delivered both face-to-face and online, such as FIBROWALK and NAT-FM, combining pain neuroscience education with therapeutic exercise and psychological components (including cognitive-behavioral approaches) alongside mindfulness practices [29,30,37].

These studies have demonstrated that a comprehensive approach combining these interventions can be highly beneficial in reducing pain and improving physical function and psychological well-being. They have also developed another project, the IMPACT Study [31], which applied acceptance and commitment therapy and behavioral activation therapy via videoconference in individuals with chronic low back pain and depressive symptoms. This intervention proved effective in improving depression, pain, QoL, and in reducing the use of pain medication.

Another noteworthy experience has been carried out in the region of Castilla y León (Spain), led by physiotherapists, combining pain neuroscience education and therapeutic physical exercise in primary care patients with chronic spinal pain. This program demonstrated significant improvements in pain perception, disability, physical function, and reductions in kinesiophobia and pain-related distress [36,38].

Our workshop, in addition to incorporating the therapies used in these cited experiences, also included other components such as healthy eating, health coaching, enhancement of self-esteem, forgiveness techniques (toward others and oneself), creative visualization, and reflection on life purpose.

Patients were selected by healthcare professionals from different centers, which may have introduced selection bias; to mitigate this bias, the professionals were informed in advance about the workshop and the target patient profile, and a preliminary interview was conducted to ensure that they met the inclusion criteria. Among the limitations of this study, it should be noted that the design (a quasi-experimental before and after study with a control group) lacked randomization, and as with any self-administered measurement scale, there is inevitably a degree of subjectivity in participants’ self-assessments.

Another limitation concerns the participant profile, which consisted predominantly of women with different underlying conditions, with a high proportion of fibromyalgia, osteoarthritis, and low back pain. This heterogeneity makes it difficult to generalize the findings to other CP conditions that were less represented in our sample. Furthermore, individuals who were unable to remain seated for several hours or who could not commit to attending all five workshop sessions were excluded; these cases were likewise excluded from the control group to avoid biasing the results.

Improvements in pain did not reach the MCID; however, MCID values are typically defined based on mean changes, and given the non-normal distribution of the data, comparisons were made using medians, which may partly explain this finding. On the other hand, this leads us to question whether this pain scale is sufficiently sensitive to detect changes in pain control, given that pain fluctuates throughout the day and varies in intensity from one day to another. For this reason, we plan to incorporate an additional outcome measure (pain catastrophizing) since, after the workshop, patients frequently report improved coping with pain, a change that is not adequately captured by the pain VAS. Among patients who did not improve, it is worth noting that the emotional state of participants was influenced not only by physical pain but also by stressful personal circumstances (family, financial, or occupational), which some reported as having hindered greater benefit. Nonetheless, many stated that the tools learned during the workshop helped them cope more effectively with subsequent challenges. A portion of participants were in the process of applying for medical leave or disability benefits, which may have affected their motivation to improve their health status. In future editions, we will attempt to identify such cases through a preliminary interview. Finally, before starting the workshop, nearly ten patients reported that they did not believe in the benefits of non-pharmacological therapies, which may have influenced the results by leading them to apply these interventions without sufficient engagement.

## 5. Conclusions

The results observed in patients in the intervention group were associated with improvements that reached or exceeded the MCID for well-being, QoL, self-perceived health status, self-esteem, anxiety, and depression; all of these benefits were maintained three months after completion of the workshop, with the exception of well-being. Statistically significant, although clinically smaller, changes that did not reach the MCID were also found for pain and resilience (both in the short and medium term) and for well-being at medium-term follow-up. In contrast, no statistically significant differences were observed in the control group for any of the outcomes, and some indicators, such as resilience and depression, even worsened.

The use of mental analgesia was associated with reduced pain in approximately three-quarters of participants, while four-fifths of the sample decreased their medication use, and the vast majority also reported improvements in lifestyle habits. In the intervention group, around two-thirds of patients indicated that they continued to manage their pain using the workshop techniques, and more than two-thirds maintained the reduction in medication use, with an increasing proportion discontinuing some drugs altogether.

The workshop demonstrated applicability across a heterogeneous patient population. These findings suggest potential benefits and underscore the need for more rigorous evaluations, including randomized clinical trials.

Further research is needed to continue evaluating the effectiveness of multicomponent programs in order to expand the therapeutic options available for the management of CP and to identify the most effective combinations of therapies for different patient profiles.

## Figures and Tables

**Table 1:** Baseline characteristics of participants in each group.

(**A**)					
		**Group**			
		**Intervention**	**Control**	Significance (*p*) *^,1^	**Total**
**Variables**		**N (%)**	**N (%)**		**N (%)**
Total participants		64 (50.0)	64 (50.0)		128 (100)
Sex	Female	58 (90.6)	57 (89.1)	0.770	115 (89.8)
Education	No formal education	1 (1.6)	1 (1.7)		2 (1.6)
	Primary	14 (22.2)	15 (25.4)		29 (23.8)
	Secondary	5 (7.9)	12 (20.3)		17 (13.9)
	High school/Vocational training	24 (38.1)	18 (30.5)		42 (34.4)
	University degree	19 (30.2)	13 (22.0)	0.311	32 (26.2)
Employment status	Full-time employment	9 (14.1)	11 (17.2)		20 (17.4)
	Part-time employment	3 (4.7)	4 (6.3)		7 (6.1)
	Working but on short-term sick leave	3 (4.7)	1 (1.6)		4 (3.5)
	Working but on long-term sick leave	21 (32.8)	22 (34.4)		43 (37.4)
	Homemaker/Not employed outside the home	22 (34.4)	19 (29.7)	0.813	41 (35.7)
Need for caregiver	No	48 (77.4)	45 (70.3)		93 (73.8)
	Yes. part-time	14 (22.6)	18 (28.1)	0.457	32 (25.4)
	Yes. full-time	0 (0)	1 (1.6)		1 (0.8)
Work absence	No	9 (14.1)	10 (15.6)		19 (14.8)
	Occasionally	26 (40.6)	19 (29.7)		45 (35.2)
	Frequently	19 (29.7)	22 (34.4)	0.625	41 (32.0)
	Not employed outside the home	10 (15.6)	13 (20.3)		23 (18.0)
Mood prior to workshop	Cheerful	3 (4.7)	1 (1.6)		4 (3.1)
	Neutral	12 (18.8)	11 (17.5)		23 (18.1)
	Discouraged	26 (40.6)	18 (28.6)	0.233	44 (34.6)
	Depressed	23 (35.9)	33 (52.4)		56 (44.1)
Generalized pain	Yes	56 (87.5)	55 (85.9)	0.795	111 (86.7)
	No	8 (12.5)	9 (14.1)		17 (13.3)
Use of analgesics	Yes	58 (90.6)	52 (81.3)		110 (85.9)
	No	6 (9.4)	10 (15.6)	0.189	16 (12.5)
	*No response*	*0 (0.0)*	*2 (3.1)*		*2 (1.6)*
Use of antidepressants, anxiolytics, or muscle relaxants	Yes	43 (67.2)	44 (68.8)		87 (68.0)
	No	21 (32.8)	18 (28.1)	0.326	39 (30.5)
	*No response*	*0 (0.0)*	*2 (3.1)*		*2 (1.6)*
History of morphine use	Yes	32 (50.0)	36 (56.3)		68 (53.1)
	No	32 (50.0)	28 (43.8)	0.479	60 (46.9)
Belief in benefits of non-pharmacological therapies	Yes	55 (85.9)	51 (79.7)		106 (82.8)
	No	9 (14.1)	13 (20.3)	0.349	22 (17.2)
(**B**)					
		**Group**			
		**Intervention**	**Control**	Significance (*p*) *^,3^	**Total**
**Scale**		**Median (IQR) ^2^**	**Median (IQR) ^2^**		**Median (IQR) ^2^**
Age		52.0 (46.3–57.0)	54.0 (46.0–57.8)	0.458	52.5 (46.0–57.0)
Maximum pain during the last 6 months	Scale 0–10	9.0 (8.0–10.0)	9.0 (8.0–10.0)	0.329	9.0 (8.0–10.0)
Pain at baseline	Scale 0–10	7.5 (6.0–8.0)	8.0 (6.0–8.8)	0.588	8.0 (6.0–8.0)
Well-being at baseline	Scale 0–10	4.0 (2.0–5.0)	4.0 (3.0–5.0)	0.178	4.0 (2.0–5.0)
Self-perceived health at baseline	Scale 0–100	40.0 (26.3–53.8)	40.0 (21.3–50.0)	0.366	40.0 (25.0–50.0)
Quality of life	Scale 0–1	0.360 (0.156–0.610)	0.354 (0.211–0.545)	0.819	0.354 (0.173–0.569)
Self-esteem	Scale 9–36	22.0 (19.0–25.8)	20.5 (18.0–24.8)	0.244	21.0 (19.0–25.0)
Resilience	Scale 6–30	17.0 (12.0–18.0)	17.0 (12.0–20.8)	0.288	17.0 (12.0–19.0)
Anxiety	Scale 0–21	13.0 (11.0–16.0)	13.0 (10.0–17.0)	0.715	13.0 (11.0–16.0)
Depression	Scale 0–21	11.0 (7.3–14.0)	11.5 (8.0–15.8)	0.549	11.0 (8.0–14.0)

* Statistical significance *p* < 0.05. ^1^ Chi-square test; ^2^ IQR: Interquartile range; ^3^ Mann–Whitney U test for independent simples.

**Table 2:** Pain location and underlying condition.

	Group				Group		
	Intervention	Control	Total		Intervention	Control	Total
	N (%)	N (%)	N (%)		N (%)	N (%)	N (%)
Disease Causing the Pain				Pain Location			
Fibromyalgia	40 (32.5)	37 (30.8)	77 (31.7)	Cervical spine	33 (8.6)	15 (4.5)	48 (6.7)
Chronic fatigue syndrome	13 (10.6)	6 (5.0)	19 (7.8)	Thoracic spine	12 (3.1)	17 (5.2)	29 (4.1)
Osteoarthritis	10 (8.1)	12 (10.0)	22 (9.1)	Lumbar spine	3 (0.8)	12 (3.6)	15 (2.1)
Back pain	5 (4.1)	7 (5.8)	12 (4.9)	Lower limbs	20 (5.2)	26 (7.9)	46 (6.5)
Low back pain	7 (5.7)	12 (10.0)	19 (7.8)	Hips	38 (9.9)	26 (7.9)	64 (9.0)
Neck pain	7 (5.7)	3 (2.5)	10 (4.1)	Pelvis	32 (8.4)	23 (7.0)	55 (7.7)
Disc herniation	6 (4.9)	9 (7.5)	15 (6.2)	Knees	22 (5.7)	12 (3.6)	34 (4.8)
Neuropathic pain	5 (4.1)	8 (6.7)	13 (5.3)	Feet	38 (9.9)	27 (8.2)	65 (9.1)
Autoimmune or neurodegenerative disease	2 (1.6)	0 (0.0)	2 (0.8)	Upper limbs	25 (6.5)	13 (3.9)	38 (5.3)
Joint pain	8 (6.5)	7 (5.8)	15 (6.2)	Shoulders	29 (7.6)	26 (7.9)	55 (7.7)
Post-traumatic pain	3 (2.4)	0 (0.0)	3 (1.2)	Hands	25 (6.5)	26 (7.9)	51 (7.2)
Migraine	4 (3.3)	2 (1.7)	6 (2.5)	Head	19 (5.0)	18 (5.5)	37 (5.2)
Other	13 (10.6)	17 (14.2)	30 (12.3)	Fibromyalgia tender points	29 (7.6)	20 (6.1)	49 (6.9)
Total	123 (100)	120 (100)	243 (100)	Chronic fatigue	27 (7.0)	37 (11.2)	64 (9.0)
				Generalized pain	16 (4.2)	28 (8.5)	44 (6.2)
				Other location	15 (3.9)	4 (1.2)	19 (2.7)
				Total	383 (100)	330 (100)	713 (100)

**Table 3:** Short-term results (at one month) Intervention group (A). Short-term results (at one month) Control group (B).

(**A**)				
**Health Indicators (Measurement Scale)**	**Initial** **Median (IQR ^1^)**	**After One Month (End of Workshop)** **Median (IQR ^1^)**	**Differences Median (IQR ^1^)**	Significance (*p*) *
Pain (0–10)	7.5 (6.0–8.0)	6.0 (4.0–7.0)	−1.3 (−3.0–0)	<0.001 *
Well-being (0–10)	4.0 (2.0–5.0)	7.0 (5.0–7.0)	3.0 (1.0–4.0)	<0.001 *
My health (0–100)	40.0 (26.3–53.8)	60.0 (49.3–75.0)	13.5 (0–30.0)	<0.001 *
Quality of life (0–1)	0.360 (0.156–0.610)	0.651 (0.477–0.821)	0.213 (0.072–0.388)	<0.001 *
Self-esteem (9–36)	22.0 (19.0–25.8)	26.0 (23.0–30.3)	4.5 (1.0–7.3)	<0.001 *
Resilience (6–30)	17.0 (12.0–18.0)	17.5 (15.0–19.3)	1.0 (−1.0–5.0)	0.004 *
Anxiety (0–21)	13.0 (11.0–16.0)	9.0 (7.0–13.0)	−3.0 (−5.0–−1.0)	<0.001 *
Depression (0–21)	11.0 (7.3–14.0)	6.0 (3.8–9.0)	−4.0 (−7.0–−2.0)	<0.001 *
(**B**)				
**Health Indicators (Measurement Scale)**	**Initial** **Median (IQR ^1^)**	**After One Month (End of Workshop)** **Median (IQR ^1^)**	**Differences** **Median (IQR ^1^)**	Significance (*p*) *
Pain (0–10)	6.0 (8.0–8.8)	7.0 (6.0–9.0)	0 (−1.0–1.0)	0.401
Well-being (0–10)	4.0 (2.0–5.0)	5.0 (2.0–7.0)	0 (−1.0–2.0)	0.325
My health (0–100)	40.0 (21.3–50.0)	45.0 (20.0–60.0)	1.5 (−10.0–20.0)	0.067
Quality of life (0–1)	0.354 (0.211–0.545)	0.344 (0.203–0.497)	0 (−0.125–0.086)	0.565
Self-esteem (9–36)	20.5 (18.0–24.8)	19.0 (16.0–24.8)	−1.0 (−2.8–0.8)	0.064
Resilience (6–30)	17.0 (12.0–20.8)	14.0 (11.0–17.0)	−1.0 (−5.0–1.0)	0.002 *
Anxiety (0–21)	13.0 (10.0–17.0)	14.5 (11.0–16.0)	1.0 (−1.0–2.0)	0.119
Depression (0–21)	11.5 (8.0–15.8)	13.0 (8.0–16.0)	1.0 (−1.0–3.0)	0.037 *

^1^ IQR: Interquartile range. * Statistical significance *p* < 0.05. Wilcoxon test.

**Table 4:** Medium-term results (four months) Intervention group (A). Medium-term results (four months) Control group (B).

(**A**)				
**Health Indicators (Measurement Scale)**	**Initial** **Median (IQR ^1^)**	**After 4 Months** **Median (IQR ^1^)**	**Differences** **Median (IQR ^1^)**	Significance (*p*) *
Pain (0–10)	7.5 (6.0–8.0)	6.0 (4.0–7.0)	−1.0 (−3.0–−1.0)	<0.001 *
Well-being (0–10)	4.0 (2.0–5.0)	5.0 (3.0–7.0)	1.0 (0–4.0)	<0.001 *
My health (0–100)	40.0 (26.3–53.8)	50.0 (40.0–70.0)	10.0 (0–30.0)	<0.001 *
Quality of life (0–1)	0.360 (0.156–0.610)	0.648 (0.384–0.722)	0.185 (0.013–0.337)	<0.001 *
Self-esteem (9–36)	22.0 (19.0–25.8)	25.0 (23.0–29.0)	3.0 (−1.0–6.0)	<0.001 *
Resilience (6–30)	17.0 (12.0–18.0)	18.0 (17.0–19.0)	1.0 (0.0–5.0)	<0.001 *
Anxiety (0–21)	13.0 (11.0–16.0)	11.0 (7.5–13.0)	−3.0 (−5.0–1.0)	<0.001 *
Depression (0–21)	11.0 (7.3–14.0)	7.0 (3.0–9.0)	−3.0 (−6.0–0)	<0.001 *
(**B**)				
**Health Indicators (Measurement Scale)**	**Initial** **Median (IQR ^1^)**	**After 4 Months Median (IQR ^1^)**	**Differences Median (IQR ^1^)**	Significance (*p*) *
Pain (0–10)	6.0 (8.0–8.8)	8.0 (7.0–9.0)	0 (−1.0–1.0)	0.290
Well-being (0–10)	4.0 (2.0–5.0)	4.5 (3.0–6.0)	0 (−1.0–1.0)	0.330
My health (0–100)	40.0 (21.3–50.0)	40.0 (30.0–53.8)	7.5 (−5.0–20.0)	0.062
Quality of life (0–1)	0.354 (0.211–0.545)	0.355 (0.140–0.583)	0 (−0.136–0.123)	0.904
Self-esteem (9–36)	20.5 (18.0–24.8)	20.0 (17.3–24.8)	0 (−2.8–2.0)	0.693
Resilience (6–30)	17.0 (12.0–20.8)	15.0 (12.0–18.8)	0 (−3.0–2.0)	0.171
Anxiety (0–21)	13.0 (10.0–17.0)	14.0 (11.0–16.0)	0 (−1.0–2.0)	0.427
Depression (0–21)	11.5 (8.0–15.8)	12.0 (8.3–16.0)	0.5 (−2.0–3.0)	0.104

^1^ IQR: Interquartile Range. * Statistical significance *p* < 0.05. Wilcoxon test.

**Table 5:** Comparison of Improvements Between the Intervention and Control Groups.

	After One Month (End of Workshop)			After 4 Months		
Health Indicators (Measurement Scale)	Intervention Group	Control Group		Intervention Group	Control Group	
	Differences Median (IQR ^1^)	Differences Median (IQR ^1^)	*p* *	Differences Median (IQR ^1^)	Differences Median (IQR ^1^)	*p* *
Pain (0–10)	−1.3 (−3.0–0)	0 (−1.0–1.0)	<0.001 *	−1.0 (−3.0–−1.0)	0 (−1.0–1.0)	<0.001 *
Well-being (0–10)	3.0 (1.0–4.0)	0 (−1.0–2.0)	<0.001 *	1.0 (0–4.0)	0 (−1.0–1.0)	0.004 *
My health (0–100)	13.5 (0–30.0)	1.5 (−10.0–20.0)	0.026 *	10.0 (0–30.0)	7.5 (−5.0–20.0)	0.047 *
Quality of life (0–1)	0.213 (0.072–0.388)	0 (−0.125–0.086)	<0.001 *	0.185 (0.013–0.337)	0 (−0.136–0.123)	<0.001 *
Self-esteem (9–36)	4.5 (1.0–7.3)	−1.0 (−2.8–0.8)	<0.001 *	3.0 (−1.0–6.0)	0 (−2.8–2.0)	<0.001 *
Resilience (6–30)	1.0 (−1.0–5.0)	−1.0 (−5.0–1.0)	<0.001 *	1.0 (0.0–5.0)	0 (−3.0–2.0)	0.001 *
Anxiety (0–21)	−3.0 (−5.0–−1.0)	1.0 (−1.0–2.0)	<0.001 *	−3.0 (−5.0–1.0)	0 (−1.0–2.0)	<0.001 *
Depression (0–21)	−4.0 (−7.0–−2.0)	1.0 (−1.0–3.0)	<0.001 *	−3.0 (−6.0–0)	0.5 (−2.0–3.0)	<0.001 *

^1^ IQR: interquartile Range. * statistical significance: *p* < 0.05. Mann–Whitney U test.

**Table 6:** Self-assessment of the workshop’s impact. Patient-Reported Outcome Measures (PROMs).

	Workshop Completed	3-Month Follow-Up
Questions	N (%)	N (%)
Pain has decreased when applying the techniques	47 (74.6)	34 (65.4)
Medication has decreased after the workshop (n = 55)	48 (81.4)	34 (68.0)
Decrease in the frequency of doses	23 (41.8)	16 (36.4)
Decrease in dosages	12 (21.8)	10 (23.8)
Switches one drug for another of a lower level	7 (12.7)	9 (21.4)
Stops taking some drugs	16 (29.1)	19 (46.3)
Did not take drugs initially	8	2
Improved Habits	58 (92.1)	40 (78.4)
Mood at the end of the workshop		
Cheerful	26 (41.3)	12 (23.1)
Neutral	24 (38.1)	23 (44.2)
Discouraged	11 (17.5)	14 (26.9)
Depressed	2 (3.2)	3 (5.8)
Total sample	63	52

## Data Availability

The data is not available due to ethical or privacy restrictions. The data presented in this study are available upon request from the corresponding author for ethical reasons of confidentiality and privacy.

## Abbreviations

The following abbreviations are used in this manuscript:

CP	Chronic Pain
QoL	Quality of life
SJDAH	San Juan de Dios del Aljarafe Hospital
PROMs	Patient-reported outcomes
PREM	Patient experience
MCID	Minimally Clinically Important Difference
SPSS	Statistical Package for Social Sciences
IQR	Interquartile range
